# Early Embryonic Chromosome Instability Results in Stable Mosaic Pattern in Human Tissues

**DOI:** 10.1371/journal.pone.0009591

**Published:** 2010-03-09

**Authors:** Hasmik Mkrtchyan, Madeleine Gross, Sophie Hinreiner, Anna Polytiko, Marina Manvelyan, Kristin Mrasek, Nadezda Kosyakova, Elisabeth Ewers, Heike Nelle, Thomas Liehr, Marianne Volleth, Anja Weise

**Affiliations:** 1 Institute of Human Genetics and Anthropology, Jena University Hospital, Jena, Germany; 2 National Medical Center “Mother and Child”, Minsk, Belarus; 3 Department of Genetics and Cytology, Yerevan State University, Yerevan, Armenia; 4 Institute of Human Genetics, Magdeburg, Germany; Ludwig-Maximilians-Universität München, Germany

## Abstract

The discovery of copy number variations (CNV) in the human genome opened new perspectives on the study of the genetic causes of inherited disorders and the aetiology of common diseases. Here, a single-cell-level investigation of CNV in different human tissues led us to uncover the phenomenon of mitotically derived genomic mosaicism, which is stable in different cell types of one individual. The CNV mosaic ratios were different between the 10 individuals studied. However, they were stable in the T lymphocytes, immortalized B lymphoblastoid cells, and skin fibroblasts analyzed in each individual. Because these cell types have a common origin in the connective tissues, we suggest that mitotic changes in CNV regions may happen early during embryonic development and occur only once, after which the stable mosaic ratio is maintained throughout the differentiated tissues. This concept is further supported by a unique study of immortalized B lymphoblastoid cell lines obtained with 20 year difference from two subjects. We provide the first evidence of somatic mosaicism for CNV, with stable variation ratios in different cell types of one individual leading to the hypothesis of early embryonic chromosome instability resulting in stable mosaic pattern in human tissues. This concept has the potential to open new perspectives in personalized genetic diagnostics and can explain genetic phenomena like diminished penetrance in autosomal dominant diseases. We propose that further genomic studies should focus on the single-cell level, to better understand the aetiology of aging and diseases mediated by somatic mutations.

## Introduction

Recent developments in the genome-wide technologies used to analyze structural variations have led to the identification of thousands of heritable copy number variations (CNV). These are submicroscopic copy number variations in DNA segments ranging from kilobases (kb) to megabases (Mb) in size, occurring in both phenotypically normal and affected subjects [1]. CNV can be a tandem or inverted duplication or may involve complex gain or loss of sequences at multiple sites within the genome [2]. It is known that some CNV can influence gene expression and play a role in the aetiology of common diseases such as diabetes, cancer, and heart disease [3], [4].

**Figure 1:**
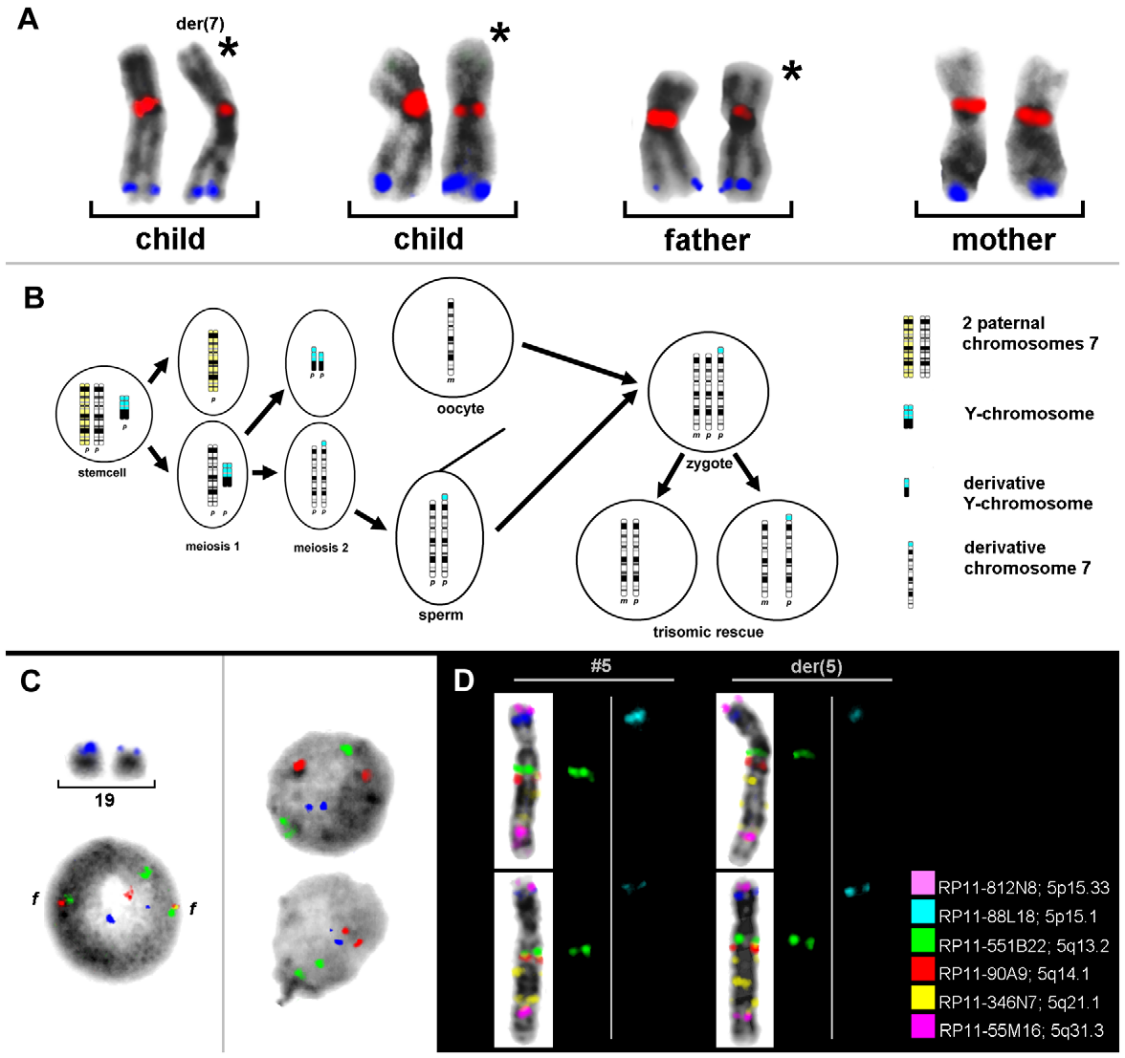
Single-cell estimation of CNV by using pod-FISH. (**A**) The parental origin of der(7) in an individual with Turner syndrome and the mosaic karyotype 45,X,der(7)t(Y;7)(p11.1∼11.2;p22.3)[122]/45,X[48] was detected with RP11-533E18 (red, 7q11.21–7q11.1) and RP11-45N9 (blue, 7q34). (**B**) The homologous chromosomes 7 of the father and der(7) of the child are marked with asterisks. The suggested development of this rare mosaic karyotype is shown schematically. (**C**) Different signal intensities for the CNV at 19p13.2 (RP11-367L15, blue) are apparent on the two homologous chromosomes 19 in metaphase, and in the interphase nuclei of all cells of an AML patient with t(8;21)(q22;q22.3) before bone-marrow transplantation (left). In the bone-marrow donor cells, 55% of the cells showed no signal intensity difference in the CNV region detected with RP11-367L15 (blue) at 19p13.2 (right, upper nucleus). However, the remaining 45% of the recipient cells showed signal intensity differences identical to those of the recipient (right, lower nucleus). As well as RP11-367L15 (blue), probes for AML1 (21q22.3, green) and ETO (8q22, red) were also applied, showing two fusion signals (f) in the patient cells. (**D**) pod-FISH using six BACs specific for different CNV regions on chromosome 5 in a patient with normal chromosome 5 and der(5) revealed different signal intensity patterns for BAC RP11-551B22 (green) and RP11-88L18 (blue).

To date, only genome-wide technologies have been available to detect such CNV and only DNA extracted from a multitude of cells could be analyzed by those approaches [1], [2], [3], [4]. Recently, two single-cell-directed approaches have been described as ‘parental-origin-determination fluorescence *in situ* hybridization’ (pod-FISH) [5] and ‘polymorphic deletion probe-based FISH’ (PDP-FISH) [6]. These techniques require CNV-region-specific bacterial artificial chromosomes (BAC; pod-FISH) and fosmid clones (PDP-FISH) to visualize copy number polymorphisms on homologous chromosomes. pod-FISH is available for 225 CNV, based on specific BAC clones of more than 150 kb in length and with variation frequencies in populations of over 10%. The selected polymorphic regions represent size variations, detectable as different signal intensities with pod-FISH [5]. In contrast, PDP-FISH has been reported for three CNV loci using fosmid probes, which distinguish signal presence and absence rather than signal intensity differences [6].

pod-FISH has already been successfully used to identify the parental origin of individual derivative chromosomes, such as the characterization of chimerism and uniparental disomy 15 [5]. However, we have found not only interindividual differences, as expected, but also intraindividual differences in the signal intensities of polymorphic BAC probes. Thus, in a woman with Turner syndrome and the mosaic karyotype 45,X,der(7)t(Y;7)(p11.1∼11.2;p22.3)[122]/45,X[48], pod-FISH determined the parental origin of the normal and derivative chromosomes 7 with two BACs (RP11-533E18 and RP11-45N9) of the 15 BACs tested [7] (Table 1 and Figure 1A). Uniparental disomy 7 was excluded for both cell lines and the result was confirmed by microsatellite analysis. Surprisingly, der(7) was found to be identical to that of the normal paternally inherited chromosome 7 in the cell line 45,X by pod-FISH, which was also confirmed with microsatellites [7]. Therefore, the evolution of that karyotype could be reconstructed (Figure 1B). A summary of the pod-FISH results for this individual showed that the number of metaphase spreads displaying different detectable signal intensities in the CNV regions ranged from 6% to 90% (Table 1), reflecting different clonal cell lines in one tissue with respect to the investigated CNV.

**Table 1:** pod-FISH analysis of der(7)t(Y;7) in an individual with Turner syndrome.

Chromosomal band	BAC	Number of metaphase spreads with different signal intensities with BAC probes [%]		
		Child	Father	Mother
7p21.1	RP11-79G16	31	11	17
7p15.3	RP11-810J17	16	12,5	38
7p15.1	RP11-643O8	70,5*	16	50
7q11.1	RP11-144H20	38	8	17
7q11.21-7q11.1	RP11-533E18	87,5**	89***	15
7q11.23	RP11-422O1	23	24	0
7q22	RP11-188C21	–	0	–
7q22.1	RP11-395B7*	6	0	0
7q22.1	RP11-344K24	6	0	18
7q31.33	RP11-807H17	50	11	38
7q32.2	RP11-537A1	21	0	0
7q33	RP11-639H21	12	16	15
7q34	RP11-45N9	6	88***	33
7q34	RP11-307I2	12	0	25
7q35	RP11-634O11	16	33	12

*Low signal intensity detected on der(7)t(Y;7) and on one normal chromosome 7 in cell line 45,X.

**Low signal intensity was always detected on der(7)t(Y;7).

***Different signal intensity levels for RP11-533E18 and RP11-45N9 in the father were observed in nonrandom combinations (on one chromosome, weak signal for RP11-533E18 accompanied by strong signal for RP11-45N9).

pod-FISH and PDP-FISH have also been applied to the analysis of cellular chimerism after bone-marrow transplantation in leukemia patients [5], [6]. To identify a low level of chimerism, it is necessary to find polymorphic regions containing CNV in 100% of the recipient and donor cells. This condition was fulfilled for the BAC clone RP11-367L15, mapping to chromosome 19p13.2, in a male suffering from *AML1–ETO*-positive acute myeloid leukemia (AML) (Figure 1C). After bone-marrow transplantation from a female donor, the cellular chimerism in the bone marrow was determined as 60% donor versus 40% recipient cells using a centromeric probe for the X chromosome. Surprisingly, no signal intensity variation for RP11-367L15 was found in only 55% of the donor cells. The remaining 45% of donor cells showed the identical pod-FISH signal pattern as those of the recipient cells (Figure 1C).

The similar intraindividual differences detected by pod-FISH turn out to be a common observation rather than an exception. Similar findings were made in a patient with cytogenetically distinguishable chromosome 5 and the derivative chromosome 5, in whom pod-FISH with six BACs specific for different CNV regions on chromosome 5 revealed different signal intensity patterns for BAC clones RP11-551B22 and RP11-88L18 on the homologous chromosomes in different metaphase spreads (Figure 1D).

For a long time it has been generally accepted that all cells in an individual are genetically identical, except in individuals with somatic mosaicism that causes disease or the rearrangements of the immunoglobulin and T-cell-receptor genes [8]. In contrast, more and more data are available demonstrating intertissular genomic variation for numerical chromosome aneuploidy contributing to mosaicism as a global mechanism for example in germ cells, placenta, human brain, skin, liver and blood [9], [10], [11]. Recently, it has been shown that extensive *de novo* and recurrent CNV occurs *in vitro* in mouse embryonic stem cell lines derived from common parental lines, leading to mosaic animals containing variants of the zygote genome [12]. A recent study of different human tissues and organs has revealed the existence of somatic CNV mosaicism [13] besides the previously reported whole chromosome aneuploidies in the above mentioned human tissues. These observations were confirmed as putative *de novo* somatic CNV events in monozygotic twins [14]. Nonetheless, somatic CNV mosaicism patterns have not yet been fully resolved, because all previous studies were performed with whole-genomic DNA extracted from a large number of cells.

To test whether different cell types have specific CNV patterns when observed at the single-cell level, we used chromosome-specific pod-FISH on metaphase spreads from 10 healthy individuals, four men and six women. Furthermore, we analysed the stability of CNV mosaicism in B-lymphoblastoid cell lines in two individuals, obtained 20 years ago and recently.

## Results and Discussion

**Figure 2:**
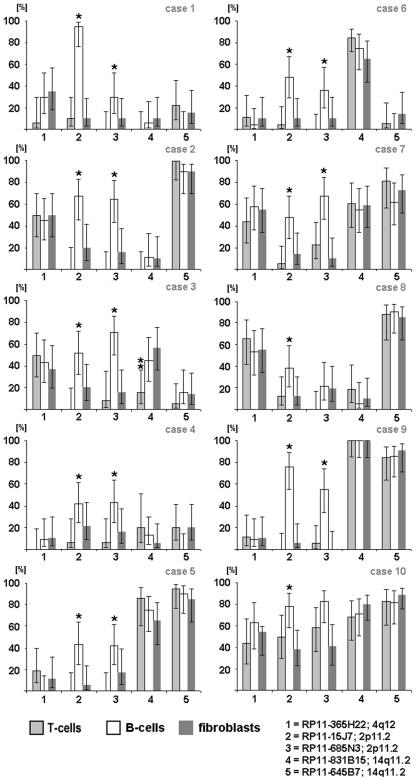
Varying signal intensity patterns of 5 selected CNV loci. Analysis of the signal intensity patterns of CNV at 2p11.2 (RP11-685N3: 88,981,161–89,122,3701bp; RP11-15J7: 88,979,594–89,141,7171bp), 4q12 (RP11-365H22: 52,354,875–52,530,859 bp), and 14q11.2 (RP11-645B7: 18,654,379–18,833,779 bp; RP11-831B15: 19,273,689–19,767,232 bp) in three cell types, each, from 10 different individuals. Notes: (1) X axis: BAC probes; Y axis: [%]  =  percentage of cells with different signal intensities. (2) In diagrams with no columns, no cells with different signal intensities for the BAC probes were detected. Abbreviations: T cells ( =  metaphases from T lymphocytes collected after PHA stimulation of the peripheral blood), B cells ( =  Epstein–Barr-virus-immortalized B-lymphoblastoid cell lines from the peripheral blood), and fibroblasts ( =  metaphases from skin biopsies). * Statistically significant difference (B cells *vs* T cells and B cells *vs* fibroblasts, *P*<0.05). ** Statistically significant difference (T cells *vs* B cells and T cells *vs* fibroblasts, *P*<0.017).

Five BACs with expected high population frequencies in the CNV regions were selected for further investigation of ten healthy individuals. Three easily accessible cell types were studied: T lymphocytes prepared from phytohemagglutinin (PHA)-stimulated peripheral blood [15], B lymphocytes from Epstein–Barr-virus-immortalized B-lymphoblastoid cell lines [16], and fibroblasts from cultivated skin biopsy samples. GTG banding analysis revealed normal karyotypes in nine individuals and a Robertsonian translocation, rob(13;15), in one subject. In one cell type, we found cells with different and equal signal intensities for the same polymorphic BAC, varying between 0% and 95%, and random variations between all 10 individuals studied (Figure 2). Surprisingly, the variation ratio within the cells of one individual remained similar in all three cell types studied (*P*>0.05; Figure 2). Only in subject 3 did the CNV located at 14q11.2 (RP11-831B15) display a nonuniform pattern in T lymphocytes *vs* that in B lymphocytes and fibroblasts (*P*<0.017; Figure 2). Also exceptions were the probes for CNV regions on chromosome 2 (RP11-15J7: 88,921,446–89,083,5701bp; RP11-685N3: 88,981,161–89,122,370 bp), which are located within the highly variable region of the immunoglobulin (Ig) gene (*IGKV*). We presume that for this reason the signal variation pattern was generally higher in the studied B cells than in other cell types (*P*<0.05). The somatic rearrangements of the Ig and T-cell-receptor (TCR) genes are well known and well characterized [17], [18]. Recombination was demonstrated by pod-FISH in T lymphocytes using BAC probes for variant CNV in the TCR gene regions (Figure S1 and Table S1).

To investigate whether once acquired CNV variation ratio maintain stable throughout life or underlie changes, we applied pod-FISH for 36 CNV regions on metaphase spreads of B-lymphoblastoid cell lines from two individuals established with a time difference of 20 years. The age of the probands was 25 and 30 years, respectively, at the first sample acquisition. As in the aforementioned study here we also found two cellular populations with different signal intensities of the same BAC, ranging from 0% to 100%. Also these varied between the two individuals studied. But interestingly, the variation ratio of BACs remained the same in the B-lymphoblastoid cell lines established with 20-year time difference within each subject (exemplified for one individual in Table S2).

The mechanism underlying the establishment of CNV mosaicism during mitosis remains unclear. The CNV-focused single cell approach applied here might only uncover a tip of the iceberg in the recently reported background of extensive chromosomal instability in human cleavage-stage embryos [19] and will help to understand how cell linage trees evolve [20]. Several studies have predicted that some CNV and nonrecurring copy number changes (CNC) in cancer cell lines, which are induced by aphidicolin or occur in some cases of Duchenne muscular dystrophy, Smith–Magenis syndrome, and Pelizaeus–Merzbacher disease, originate from nonhomologous end joining, fork stalling and template switching, or microhomology/microsatellite-induced replication mechanisms [21], [22]. Moreover, such evolutionarily significant hotspots as fragile sites and aphidicolin-induced CNC might resemble many human CNV [21] (Table S3).

**Figure 3:**
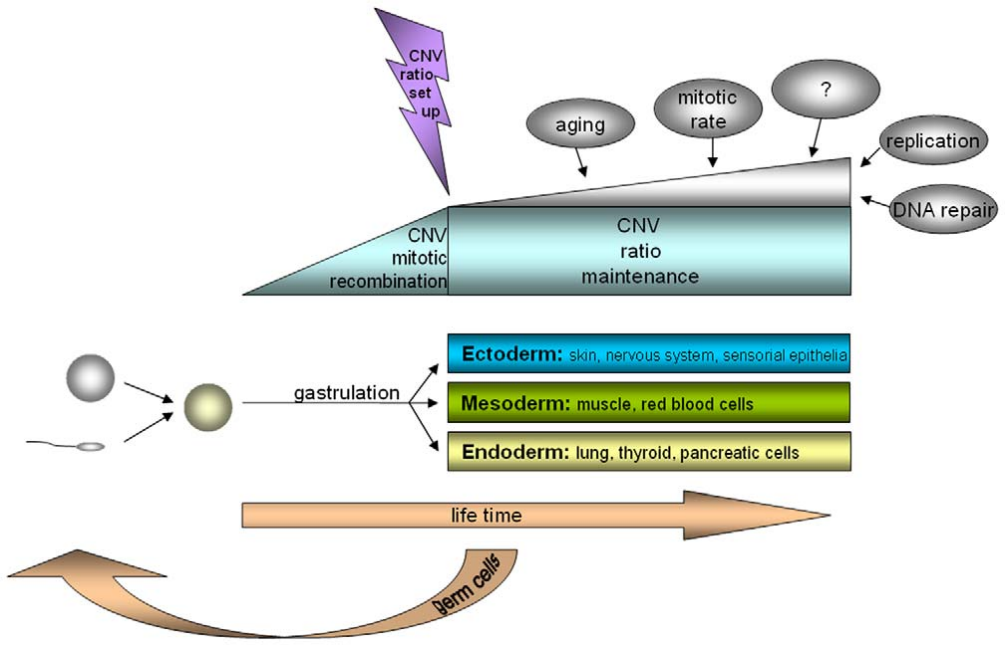
Scheme for the development of somatic CNV mosaicism. Parental germ cells with a certain haploid pattern of CNV fuse to a zygote with a defined diploid pattern of CNV. It is hypothesized that in early embryogenesis, the mitotic recombination of single CNV occurs, leading to a somatic mosaicism in the CNV pattern, which is stable until at least the end of gastrulation and the formation of the germ layers. The ratio of this CNV mosaicism is stable over a lifetime and across different cell types, except when other factors directly influence DNA stability.

Overall, we provide the first sound evidence of somatic mosaicism for CNV, with stable variation ratios in different cell types. We have described at least two different cell lines: one with the same or equal signal intensity and one with a significant signal intensity difference in the investigated CNV region on homologous chromosomes. The CNV ratios of these cell lines differ between individuals but not within individuals. We suggest that the somatic recombination of polymorphic regions might occur at a relatively early time point in embryogenesis because all the well-differentiated cells studied have similar CNV mosaic patterns (Figure 3). This hypothesis is substantiated by new findings of complex chromosomal imbalances involving not only whole chromosomal aneuploidies and uniparental disomies but also segmental deletions, duplications, and amplifications in human cleavage-stage *in vitro*-fertilized embryos [19]. Interestingly, when a CNV pattern is once established, the variation ratio seems to be stable throughout all tissues and over a life span of 20 years minimum studied here; or varies, as in the case of the CNV in T cells compared with the other cell types studied. Finally, the study of this phenomenon should open new perspectives in personalized genetic diagnostics. The intraindividual specific mosaicism ratio at a certain susceptibility gene for a disease, as reported here, might have a higher impact than previously expected, especially for so-called ‘multi-factorial diseases’, and might also explain clinical genetic phenomena like diminished penetrance in autosomal dominant diseases or clinical signs without apparent mutations when only a single tissue is screened.

## Materials and Methods

### Material

The study included heparinised peripheral blood from an individual with der(7)t(Y,7) and both parents; a mentally retarded patient with der(5)(pter->q21::q23->q21::q21->qter) and 10 healthy individuals, as well as unstimulated bone-marrow samples from an AML patient, and Epstein–Barr-virus-immortalized lymphoblastoid cell lines established from EDTA-treated blood and biopsy samples derived from swages of upper arm skin from the 10 healthy individuals. The age of these later mentioned 10 probands ranged from 23 to 31 years, with a median age of 26 years and a male:female ratio of 4:6. All participants involved in this study gave a written informed consent. The experimental procedures performed on human tissue samples, the establishment of Epstein–Barr-virus- (EBV) immortalized B-lymphoblastoid cell lines and the consent form was approved by the Ethics Committee of Friedrich Schiller University Hospital Jena (internal code 1457-12/04). Also EBV immortalized B-lymphoblastoid cell lines from the same female individual were established at age of 25 and 45 and from one male at 30 and 50 years, respectively.

### Cytogenetics

Metaphase chromosome preparations were obtained from PHA-stimulated peripheral blood according to standard techniques [23]. Cytogenetic analyses of all samples were performed using GTG banding. The karyotypes were described according to the International System for Human Cytogenetic Nomenclature (ISCN, 2009) [24].

### BAC Clone Selection for pod-FISH

BAC clones from CNV regions were selected by the “Database of Genomic Variants” (http://projects.tcag.ca/variation/), purchased from the Children's Hospital Oakland Research Institute (CHORI), Oakland, CA, USA, or kindly provided by the Sanger Centre, UK. All BAC DNA was isolated, PCR amplified, and labelled by nick translation (Roche, Karlsruhe, Germany) [5].

### Chromosome Specific pod-FISH

Three chromosome-specific probe sets for chromosome 7, altogether containing 15 BAC probes, were applied to the samples from the subject with der(7)t(Y,7) and the parental samples (Table 1).

An LSI dual-color AML1/ETO probe (Abbott Molecular, USA) was applied to the samples from the AML patient, according to manufacturer's instructions, together with BAC RP11-367L15 (19p13.2) according to standard procedures. One hundred nuclei were analyzed for each bone-marrow sample.

The chromosome-specific pod-FISH set for chromosome 5 (RP11-812N8, 5p15.33; RP11-88L18, 5p15.1; RP11-551B22, 5q13.2; RP11-90A9, 5q14.1; RP11-346N7, 5q21.1; RP11-55M16, 5q31.3) was applied to the samples from the individual with der(5).

An all-chromosome-directed pod-FISH probe set (225 BACs) was applied to analyze the PHA-stimulated peripheral-blood T lymphocytes from subject 10. Five BACs (RP11-15J7, 2p11.2; RP11-685N3, 2p11.2; RP11-365H22, 4q12; RP11-831B15, 14q11.2; RP11-645B7, 14q11.1) were chosen for application to the samples from the 10 healthy individuals. All pod-FISH sets were applied and evaluated on 20–30 metaphase spreads, as previously reported [5]. Statistical analysis was performed using the confidence interval of a proportion, one-way analysis of variance and the all-pairwise multiple comparison procedure (Holm–Sidak method). Statistical significance was defined as P<0.05.

## Supporting Information

Figure S1 pod-FISH within the variable T-cell receptor beta locus in chromosomal region 7q34. Fluorescence in situ hybridization with four BAC probes (RP11-1141E10 [green], RP11-7P7 [red], RP11-466C10 [purple], RP11-157N15 [blue]) located in the variable T-cell receptor beta locus in chromosomal region 7q34 revealed different signal constellations in phytohemagglutinin (PHA)-stimulated peripheral blood and umbilical cord blood. A) Deletion of RP11-1141E10 and partial deletion of RP11-466C10. B) The simultaneous deletion of RP11-1141E10, RP11-7P7, and RP11-466C10. C) No deletion in the 7q34 region was visualized by FISH.
(0.35 MB TIF)

Table S1 FISH analysis of the variable T-cell receptor beta locus region using BAC DNA probes. * FISH analysis of BACs was not performed in theses cases. ** Metaphase spreads [%] without probe signal on both homologous chromosomes and with probe signal on only one homologous chromosome. *** About 80 kb of BAC RP11-157N15 covers the T-cell receptor beta variable (TRBV) gene, 130 kb are located proximal. We found the BAC signal on both homologous chromosomes 7 in all the metaphase spreads studied. However, in ∼50% of the cells, a different signal intensity was observed on homologous chromosome. Start/Stop[kb]: Start/Stop of BAC clones are with respect to the UCSC genome browser, version March 2006. T cells from PB: T lymphocytes from the PHA-stimulated peripheral blood of three healthy probands. B cells: B lymphocytes from Epstein-Barr-virus-immortalized B-lymphoblastoid cell lines from three healthy probands. AF: Suspensions from the amniotic fluid of two probands. T cells from UCB: T lymphocytes from PHA-stimulated umbilical cord blood (UCB) from four probands. Maternal contamination was excluded by the Kleihauer-Betke test. AML patient after BMT: A bone-marrow suspension from a patient with acute myeloid leukemia (AML) after bone-marrow transplantation (BMT). CML patient after BMT: A bone-marrow suspension from a patient with chronic myeloid leukemia (CML) after BMT. B-ALL cases: Bone-marrow suspensions from patients with B-cell acute lymphoblastic leukemia (B-ALL). T-ALL cases (BM): Bone-marrow suspensions of patients with T-cell ALL (T-ALL). T-ALL patient (PB): Peripheral blood suspension from a patient with T-ALL. Sperm: Acetic acid-methanol-fixed sperm. Because of the haploidy of the spermatozoids, there was only one signal identified for each BAC in 100% of the cells. No deletion was found.
(0.12 MB DOC)

Table S2 Results of pod-FISH analysis of B-lymphoblastoid cell lines from subject 1 established with a time interval of 20 years. T-test (P = <0,01) was applied. To test for statistic significance.
(0.17 MB DOC)

Table S3 Sequence-mapped fragile sites resemble human copy number variations (CNV).
(0.13 MB DOC)
