# Supplementary figures and images for: Early Embryonic Chromosome Instability Results in Stable Mosaic Pattern in Human Tissues
Source: PLoS One. 2010 Mar 9;5(3):e9591. doi: 10.1371/journal.pone.0009591 (PMC2834743; doi:10.1371/journal.pone.0009591)

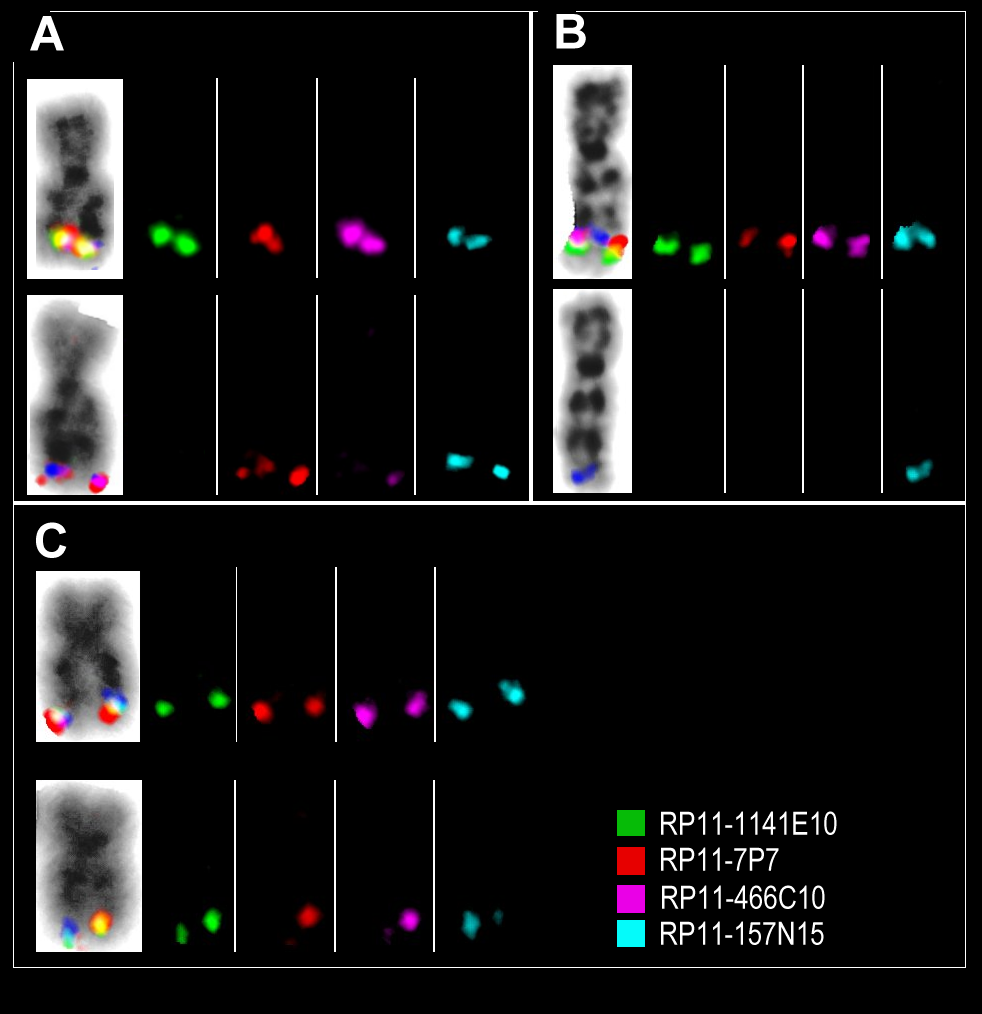

Supplement: Figure S1 — pod-FISH within the variable T-cell receptor beta locus in chromosomal region 7q34. Fluorescence in situ hybridization with four BAC probes (RP11-1141E10 [green], RP11-7P7 [red], RP11-466C10 [purple], RP11-157N15 [blue]) located in the variable T-cell receptor beta locus in chromosomal region 7q34 revealed different signal constellations in phytohemagglutinin (PHA)-stimulated peripheral blood and umbilical cord blood. A) Deletion of RP11-1141E10 and partial deletion of RP11-466C10. B) The simultaneous deletion of RP11-1141E10, RP11-7P7, and RP11-466C10. C) No deletion in the 7q34 region was visualized by FISH. (0.35 MB TIF) [file pone.0009591.s001.tif]
