# Supplementary material for: Early Embryonic Chromosome Instability Results in Stable Mosaic Pattern in Human Tissues
Source: PLoS One. 2010 Mar 9;5(3):e9591. doi: 10.1371/journal.pone.0009591 (PMC2834743; doi:10.1371/journal.pone.0009591)
Supplement: Table S1 — FISH analysis of the variable T-cell receptor beta locus region using BAC DNA probes. * FISH analysis of BACs was not performed in theses cases. ** Metaphase spreads [%] without probe signal on both homologous chromosomes and with probe signal on only one homologous chromosome. *** About 80 kb of BAC RP11-157N15 covers the T-cell receptor beta variable (TRBV) gene, 130 kb are located proximal. We found the BAC signal on both homologous chromosomes 7 in all the metaphase spreads studied. However, in ∼50% of the cells, a different signal intensity was observed on homologous chromosome. Start/Stop[kb]: Start/Stop of BAC clones are with respect to the UCSC genome browser, version March 2006. T cells from PB: T lymphocytes from the PHA-stimulated peripheral blood of three healthy probands. B cells: B lymphocytes from Epstein-Barr-virus-immortalized B-lymphoblastoid cell lines from three healthy probands. AF: Suspensions from the amniotic fluid of two probands. T cells from UCB: T lymphocytes from PHA-stimulated umbilical cord blood (UCB) from four probands. Maternal contamination was excluded by the Kleihauer-Betke test. AML patient after BMT: A bone-marrow suspension from a patient with acute myeloid leukemia (AML) after bone-marrow transplantation (BMT). CML patient after BMT: A bone-marrow suspension from a patient with chronic myeloid leukemia (CML) after BMT. B-ALL cases: Bone-marrow suspensions from patients with B-cell acute lymphoblastic leukemia (B-ALL). T-ALL cases (BM): Bone-marrow suspensions of patients with T-cell ALL (T-ALL). T-ALL patient (PB): Peripheral blood suspension from a patient with T-ALL. Sperm: Acetic acid-methanol-fixed sperm. Because of the haploidy of the spermatozoids, there was only one signal identified for each BAC in 100% of the cells. No deletion was found. (0.12 MB DOC) [file pone.0009591.s002.doc]

**Table S1**

FISH analysis of the variable T-cell receptor beta locus region using BAC DNA probes

| Chromosomal band | BAC | Start/  Stop  [kb] | Metaphase spreads with probe signal on only one homologous chromosome [%] | | | | | | | | | | | | | | | | | | | |
| --- | --- | --- | --- | --- | --- | --- | --- | --- | --- | --- | --- | --- | --- | --- | --- | --- | --- | --- | --- | --- | --- | --- |
|  |  |  | T cells  from PB | | | B cells | | | AF | | T cells from UCB | | | | AML patient after BMT | CML  patient after BMT | B-ALL patients (BM) | | T-ALL patients (BM) | | T-ALL (PB) | Sperm |
| 7q34 | RP11-786A19 | 141.367/ | 0 | 0 | 0 | -* | - | - | - | - | - | - | - | - | 0 | 0 | 0 | 0 | 0 | 0 | 0 | 100 |
|  |  | 141.547 |  |  |  |  |  |  |  |  |  |  |  |  |  |  |  |  |  |  |  |  |
| 7q34 | RP11-1141E10 | 141.610/ | 50 | 42 | 47 | 0 | 0 | 0 | 0 | 0 | 66 | 61 | 63 | 52 | 0 | 0 | 0 | 0 | 0 | 0 | 0 | 100 |
|  |  | 142.240 |  |  |  |  |  |  |  |  |  |  |  |  |  |  |  |  |  |  |  |  |
| 7q34 | RP11-7P7 | 141.818/ | 47 | 40 | 47 | 0 | 0 | 0 | 0 | 0 | 33 | 56 | 42 | 11 | 0 | 0 | 0 | 0 | 0 | 0 | 0 | 100 |
|  |  | 141. 973 |  |  |  |  |  |  |  |  |  |  |  |  |  |  |  |  |  |  |  |  |
| 7q34 | RP11-466C10 | 142.146/ | 56 | 38 | 57 | 0 | 0 | 0 | 0 | 0 | 59 | 56 | 58 | 47 | 0 | 0 | 0 | 0 | 0 | 0 | 0 | 100 |
|  |  | 142.147 |  |  |  |  |  |  |  |  |  |  |  |  |  |  |  |  |  |  |  |  |
| 7q34 | RP11-157N15*** | 142.143/ | 0 | 0 | 0 | 0 | 0 | 0 | 0 | 0 | 0 | 0 | 0 | 0 | 0 | 0 | 0 | 0 | 0 | 0 | 0 | 100 |
|  |  | 142.323 |  |  |  |  |  |  |  |  |  |  |  |  |  |  |  |  |  |  |  |  |
| 14q11.2 | RP11-246A2 | 21.636/ | 83** | 62** | 80** | - | - | - | - | - | - | - | - | - | 0 | 0 | 0 | 0 | 0 | 0 | 0 | 100 |
|  |  | 21.813 |  |  |  |  |  |  |  |  |  |  |  |  |  |  |  |  |  |  |  |  |
| 14q11.2 | RP11-614K19 | 21.726/ | 100** | 94** | 82** | - | - | - | - | - | - | - | - | - | 0 | 0 | 0 | 0 | 0 | 0 | 0 | 100 |
|  |  | 21.915 |  |  |  |  |  |  |  |  |  |  |  |  |  |  |  |  |  |  |  |  |
| 14q11.2 | RP11-1083M21 | 22.138/ | 0 | 0 | 0 | - | - | - | - | - | - | - | - | - | 0 | 0 | 0 | 0 | 0 | 0 | 0 | 100 |
|  |  | 22.343 |  |  |  |  |  |  |  |  |  |  |  |  |  |  |  |  |  |  |  |  |
| 14q11.2 | RP11-95A20 | 22.141/ | 0 | 0 | 0 | - | - | - | - | - | - | - | - | - | 0 | 0 | 0 | 0 | 0 | 0 | 0 | 100 |
|  |  | 22.343 |  |  |  |  |  |  |  |  |  |  |  |  |  |  |  |  |  |  |  |  |
