# Supplementary material for: Early Embryonic Chromosome Instability Results in Stable Mosaic Pattern in Human Tissues
Source: PLoS One. 2010 Mar 9;5(3):e9591. doi: 10.1371/journal.pone.0009591 (PMC2834743; doi:10.1371/journal.pone.0009591)
Supplement: Table S2 — Results of pod-FISH analysis of B-lymphoblastoid cell lines from subject 1 established with a time interval of 20 years. T-test (P = <0,01) was applied. To test for statistic significance. (0.17 MB DOC) [file pone.0009591.s003.doc]

**Table S2**

| CNV-BAC | 1989 | | | 2009 | | | Statistically significant difference between  1989 and 2009 |
| --- | --- | --- | --- | --- | --- | --- | --- |
|  | AA (%) | AB (%) | Statistically significant difference between  AA and AB | AA (%) | AB (%) | Statistically significant difference between  AA and AB |  |
| RP11-812N8 | 86,4 | 13,6 | YES | 81,0 | 19,0 | YES | NO |
| RP11-813J10 | 100,0 | 0,0 | YES | 81,0 | 19,0 | YES | NO |
| RP11-461G12 | 81,8 | 18,2 | YES | 94,7 | 5,3 | YES | NO |
| RP11-567A12 | 95,5 | 4,5 | YES | 94,1 | 5,9 | YES | NO |
| RP11-1143N16 | 95,5 | 4,5 | YES | 95,2 | 4,8 | YES | NO |
| RP11-767D22 | 90,9 | 9,1 | YES | 100,0 | 0,0 | YES | NO |
| RP11-79G16 | 84,0 | 16,0 | YES | 73,9 | 26,1 | YES | NO |
| RP11-533E18 | 28,0 | 72,0 | YES | 8,3 | 91,7 | YES | YES  (P = 0,008) |
| RP11-188C21 | 84,0 | 16,0 | YES | 82,6 | 17,4 | YES | NO |
| RP11-807H17 | 100,0 | 0,0 | YES | 91,7 | 8,3 | YES | NO |
| RP11-45N9 | 84,0 | 16,0 | YES | 82,6 | 17,4 | YES | NO |
| RP11-810J17 | 100,0 | 0,0 | YES | 88,9 | 11,1 | YES | NO |
| RP11-144H20 | 85,7 | 14,3 | YES | 77,8 | 22,2 | YES | NO |
| RP11-395B7* | 100,0 | 0,0 | YES | 96,3 | 3,7 | YES | NO |
| RP11-537A1 | 100,0 | 0,0 | YES | 96,3 | 3,7 | YES | NO |
| RP11-1141E10 | 100,0 | 0,0 | YES | 100,0 | 0,0 | YES | NO |
| RP11-634O11 | 90,5 | 9,5 | YES | 88,9 | 11,1 | YES | NO |
| RP11-643O8 | 100,0 | 0,0 | YES | 90,0 | 10,0 | YES | NO |
| RP11-422O1 | 100,0 | 0,0 | YES | 100,0 | 0,0 | YES | NO |
| RP11-344K24 | 100,0 | 0,0 | YES | 100,0 | 0,0 | YES | NO |
| RP11-639H21 | 100,0 | 0,0 | YES | 100,0 | 0,0 | YES | NO |
| RP11-307I2 | 100,0 | 0,0 | YES | 100,0 | 0,0 | YES | NO |
| RP11-645B7 | 5,0 | 95,0 | YES | 0,0 | 100,0 | YES | NO |
| RP11-642H22 | 100,0 | 0,0 | YES | 100,0 | 0,0 | YES | NO |
| RP11-350H11 | 100,0 | 0,0 | YES | 100,0 | 0,0 | YES | NO |
| RP11-1A3 | 100,0 | 0,0 | YES | 100,0 | 0,0 | YES | NO |
| RP11-831B15 | 100,0 | 0,0 | YES | 100,0 | 0,0 | YES | NO |
| RP11-125A5 | 100,0 | 0,0 | YES | 100,0 | 0,0 | YES | NO |
| RP3-414A15 | 100,0 | 0,0 | YES | 100,0 | 0,0 | YES | NO |
| RP11-160E2 | 55,0 | 45,0 | NO | 41,2 | 58,8 | NO | NO |
| RP11-678G7 | 100,0 | 0,0 | YES | 100,0 | 0,0 | YES | NO |
| RP11-121G16 | 100,0 | 0,0 | YES | 100,0 | 0,0 | YES | NO |
| RP11-730A9 | 75,0 | 25,0 | YES | 94,1 | 5,9 | YES | NO |
| RP11-79F15 | 71,4 | 28,6 | YES | 45,5 | 54,5 | NO | NO |
| RP11-1096L2 | 95,2 | 4,8 | YES | 95,2 | 4,8 | YES | NO |
| RP11-21O13 | 100,0 | 0,0 | YES | 100,0 | 0,0 | YES | NO |

| CNV-BAC | 1989 | | | 2009 | | | Statistically significant difference between  1989 and 2009 |
| --- | --- | --- | --- | --- | --- | --- | --- |
|  | AA (%) | AB (%) | Statistically significant difference between  AA and AB | AA (%) | AB (%) | Statistically significant difference between  AA and AB |  |
| RP11-812N8 | 90,5 | 9,5 | Yes | 84,2 | 15,8 | Yes | NO |
| RP11-813J10 | 90,5 | 9,5 | Yes | 94,7 | 5,3 | Yes | NO |
| RP11-461G12 | 95,2 | 4,8 | Yes | 84,2 | 15,8 | Yes | NO |
| RP11-567A12 | 81,0 | 19,0 | Yes | 94,7 | 5,3 | Yes | NO |
| RP11-1143N16 | 95,2 | 4,8 | Yes | 100,0 | 0,0 | Yes | NO |
| RP11-767D22 | 95,2 | 4,8 | Yes | 100,0 | 0,0 | Yes | NO |
| RP11-77P12 | 90,0 | 10,0 | Yes | 100,0 | 0,0 | Yes | NO |
| RP11-813M23 | 95,0 | 5,0 | Yes | 95,5 | 4,5 | Yes | NO |
| RP11-44F1 | 95,0 | 5,0 | Yes | 100,0 | 0,0 | Yes | NO |
| RP11-133G21 | 83,3 | 16,7 | Yes | 90,9 | 9,1 | Yes | NO |
| RP11-265K23 | 100,0 | 0,0 | Yes | 95,5 | 4,5 | Yes | NO |
| RP11-79G16 | 100,0 | 0,0 | Yes | 95,2 | 4,8 | Yes | NO |
| RP11-533E18 | 100,0 | 0,0 | Yes | 95,5 | 4,5 | Yes | NO |
| RP11-188C21 | 100,0 | 0,0 | Yes | 100,0 | 0,0 | Yes | NO |
| RP11-807H17 | 100,0 | 0,0 | Yes | 90,9 | 9,1 | Yes | NO |
| RP11-45N9 | 100,0 | 0,0 | Yes | 100,0 | 0,0 | Yes | NO |
| RP11-810J17 | 100,0 | 0,0 | Yes | 100,0 | 0,0 | Yes | NO |
| RP11-144H20 | 100,0 | 0,0 | Yes | 100,0 | 0,0 | Yes | NO |
| RP11-395B7 | 100,0 | 0,0 | Yes | 100,0 | 0,0 | Yes | NO |
| RP11-537A1 | 100,0 | 0,0 | Yes | 100,0 | 0,0 | Yes | NO |
| RP11-1141E10 | 81,8 | 18,2 | Yes | 100,0 | 0,0 | Yes | NO |
| RP11-634O11 | 95,5 | 4,5 | Yes | 100,0 | 0,0 | Yes | NO |
| RP11-645B7 | 100,0 | 0,0 | Yes | 90,0 | 10,0 | Yes | NO |
| RP11-642H22 | 100,0 | 0,0 | Yes | 100,0 | 0,0 | Yes | NO |
| RP11-350H11 | 100,0 | 0,0 | Yes | 100,0 | 0,0 | Yes | NO |
| RP11-1A3 | 100,0 | 0,0 | Yes | 100,0 | 0,0 | Yes | NO |
| RP11-831B15 | 78,9 | 21,1 | Yes | 61,1 | 38,9 | NO | NO |
| RP11-125A5 | 100,0 | 0,0 | Yes | 94,4 | 5,6 | Yes | NO |
| RP3-414A15 | 100,0 | 0,0 | Yes | 94,4 | 5,6 | Yes | NO |
| RP11-160E2 | 80,0 | 20,0 | Yes | 100,0 | 0,0 | Yes | NO |
| RP11-678G7 | 82,4 | 17,6 | Yes | 89,5 | 10,5 | Yes | NO |
| RP11-121G16 | 23,5 | 76,5 | Yes | 21,1 | 78,9 | Yes | NO |
| RP11-730A9 | 81,8 | 18,2 | Yes | 93,8 | 6,3 | Yes | NO |
| RP11-79F15 | 76,3 | 23,7 | Yes | 75,4 | 24,6 | Yes | NO |
| RP11-1096L2 | 100,0 | 0,0 | Yes | 95,0 | 5,0 | Yes | NO |
| RP11-21O13 | 100,0 | 0,0 | Yes | 85,0 | 15,0 | Yes | NO |
