# Supplementary material for: Early Embryonic Chromosome Instability Results in Stable Mosaic Pattern in Human Tissues
Source: PLoS One. 2010 Mar 9;5(3):e9591. doi: 10.1371/journal.pone.0009591 (PMC2834743; doi:10.1371/journal.pone.0009591)
Supplement: Table S3 — Sequence-mapped fragile sites resemble human copy number variations (CNV). (0.13 MB DOC) [file pone.0009591.s004.doc]

**Table S3**

| FS | Mapping | Mb | Size | CNV/ | References |
| --- | --- | --- | --- | --- | --- |
|  | Cyto | start/stop | Mb | INDEL |  |
| FRA1E | 1p21.3 | 97.521 | 0.411 | x | (*S1*) |
|  | 1p21.3 | 97.932 |  |  |  |
| FRA1H* | 1q41 | 214.175 | 10.634 | x/x | (*S2*) |
|  | 1q42.12 | 224.809 |  |  |  |
| FRA2G* | 2q24.3 | 169.207 | 0.973 | x | (*S3*) |
|  | 2q31.1 | 170.180 |  |  |  |
| FRA4F | 4q22.1 | 89.514 | 8.018 | x | (*S4*) |
|  | 4q22.3 | 97.532 |  |  |  |
| FRA5C | 5q31.1 | 133.085 | 1.389 | x | (*S5, S6*) |
|  | 5q31.1 | 134.474 |  |  |  |
| FRA6H* | 6p22.1 | 27.912 | 9.350 | x | (*S9*) |
|  | 6p21.2 | 37.262 |  |  |  |
| FRA6F | 6q21 | 111.685 | 0.841 | x | (*S8*) |
|  | 6q21 | 112.526 |  |  |  |
| FRA6E* | 6q25.3 | 160.097 | 3.541 | x | (*S9*) |
|  | 6q26 | 163.638 |  |  |  |
| FRA7E | 7q21.11 | 80.295 | 4.432 | x | (*S10*) |
|  | 7q21.11 | 84.727 |  |  |  |
| FRA7K | 7q31.1 | 110.439 | 0.453 | x | (*S11*) |
|  |  | 110.892 |  |  |  |
| FRA7G | 7q31.1 | 112.017 | 4.209 | x | (*S12*) |
|  | 7q31.2 | 116.226 |  |  |  |
| FRA7I | 7q35 | 144.399 | 1.383 | x | (*S13*) |
|  | 7q35 | 145.782 |  |  |  |
| FRA9G* | 9p22.2 | 17.136 | 0.353 | x | (*S14*) |
|  | 9p22.2 | 17.489 |  |  |  |
| FRA9E* | 9q31.2 | 108.394 | 9.687 | x | (*S15*) |
|  | 9q33.1 | 118.081 |  |  |  |
| FRA10D | 10q22.3 | 79.688 | 0.157 | x | (*S6*) |
|  |  | 79.845 |  |  |  |
| FRA10E | 10q25.2 | 112.963 | 0.174 | – | (*S10*) |
|  |  | 113.137 |  |  |  |
| FRA11E | 11p13 | 31.922 | 2.063 | x | (*S16*) |
|  | 11p13 | 33.985 |  |  |  |
| FRA11F* | 11q14.1 | 83.932 | 7.438 | x | (*S17*) |
|  | 11q14.3 | 91.370 |  |  |  |
| FRA11G* | 11q23.2 | 113.120 | 4.549 | x | (*S7*) |
|  | 11q23.3 | 117.669 |  |  |  |
| FRA13A* | 13q13.2 | 34.444 | 0.637 | x | (*S18*) |
|  | 13q13.3 | 35.081 |  |  |  |
| FRA13E* | 13q22.1 | 72.184 | 3.100 | x | (*S7*) |
|  | 13q22.3 | 75.284 |  |  |  |
| FRA16D | 16q23.1 | 76.596 | 0.983 | x | (*S19*) |
|  | 16q23.1 | 77.579 |  |  |  |

| FS | Mapping | Mb | Size | CNV/ | References |
| --- | --- | --- | --- | --- | --- |
|  | Cyto | start/stop | Mb | INDEL |  |
| FRA18A | 18q12.2 | 32.376 | 0.177 | x | (*S5*) |
|  |  | 32.553 |  |  |  |
| FRA18C | 18q22.2 | 65.506 | 0.155 | x | (*S20*) |
|  |  | 65.661 |  |  |  |
| FRAXB* | Xp22.31 | 6.605 | 0.830 | x | (*S21*) |
|  |  | 7.435 |  |  |  |

* Exact proximal and distal breakpoint of fragile sites mapped

FS: Fragile site

Size Mb: Size in Mb with respect to the UCSC genome browser, version March 2006

**References**

S1. Hormozian F, Schmitt JG, Sagulenko E, Schwab M, Savelyeva L (2007) [FRA1E common fragile site breaks map within a 370kilobase pair region and disrupt the dihydropyrimidine dehydrogenase gene (DPYD).](http://www.ncbi.nlm.nih.gov/pubmed/16556484?itool=EntrezSystem2.PEntrez.Pubmed.Pubmed_ResultsPanel.Pubmed_RVDocSum&ordinalpos=2) Cancer Lett 246: 82-91.

S2. Curatolo A, Limongi ZM, Pelliccia F, Rocchi A (2007) [Molecular characterization of the human common fragile site FRA1H.](http://www.ncbi.nlm.nih.gov/pubmed/17311248?itool=EntrezSystem2.PEntrez.Pubmed.Pubmed_ResultsPanel.Pubmed_RVDocSum&ordinalpos=2) Genes Chromosomes Cancer 46: 487-493.

S3. Limongi MZ, Pelliccia F, Rocchi A (2003) [Characterization of the human common fragile site FRA2G.](http://www.ncbi.nlm.nih.gov/pubmed/12620385?itool=EntrezSystem2.PEntrez.Pubmed.Pubmed_ResultsPanel.Pubmed_RVDocSum&ordinalpos=4) Genomics 81: 93-97.

# S4. [Rozier L](http://www.ncbi.nlm.nih.gov/pubmed?term="Rozier L"%5BAuthor%5D&itool=EntrezSystem2.PEntrez.Pubmed.Pubmed_ResultsPanel.Pubmed_RVAbstract), [El-Achkar E](http://www.ncbi.nlm.nih.gov/pubmed?term="El-Achkar E"%5BAuthor%5D&itool=EntrezSystem2.PEntrez.Pubmed.Pubmed_ResultsPanel.Pubmed_RVAbstract), [Apiou F](http://www.ncbi.nlm.nih.gov/pubmed?term="Apiou F"%5BAuthor%5D&itool=EntrezSystem2.PEntrez.Pubmed.Pubmed_ResultsPanel.Pubmed_RVAbstract), [Debatisse M](http://www.ncbi.nlm.nih.gov/pubmed?term="Debatisse M"%5BAuthor%5D&itool=EntrezSystem2.PEntrez.Pubmed.Pubmed_ResultsPanel.Pubmed_RVAbstract) (2004) Characterization of a conserved aphidicolin-sensitive common fragile site at human 4q22 and mouse 6C1: possible association with an inherited disease and cancer. Oncogene 23: 6872-6880.

S5. Ruiz-Herrera A, Garcia F, Frönicke L, Ponsà M, Egozcue J et al. (2004) [Conservation of aphidicolin-induced fragile sites in Papionini (Primates) species and humans.](http://www.ncbi.nlm.nih.gov/pubmed/15505403?itool=EntrezSystem2.PEntrez.Pubmed.Pubmed_ResultsPanel.Pubmed_RVDocSum&ordinalpos=5) Chromosome Res 12: 683-690.

S6. Thorland EC, Myers SL, Gostout BS, Smith DI (2003) [Common fragile sites are preferential targets for HPV16 integrations in cervical tumors.](http://www.ncbi.nlm.nih.gov/pubmed/12606949?itool=EntrezSystem2.PEntrez.Pubmed.Pubmed_ResultsPanel.Pubmed_RVDocSum&ordinalpos=1) Oncogene 22: 1225-1237.

S7. Fechter A, Buettel I, Kuehnel E, Savelyeva L, Schwab M (2007) [Common fragile site FRA11G and rare fragile site FRA11B at 11q23.3 encompass distinct genomic regions.](http://www.ncbi.nlm.nih.gov/pubmed/17063465?itool=EntrezSystem2.PEntrez.Pubmed.Pubmed_ResultsPanel.Pubmed_RVDocSum&ordinalpos=2) Genes Chromosomes Cancer 46: 98-106.

S8. Morelli C, Karayianni E, Magnanini C, Mungall AJ, Thorland E et al. (2002) [Cloning and characterization of the common fragile site FRA6F harboring a replicative senescence gene and frequently deleted in human tumors.](http://www.ncbi.nlm.nih.gov/pubmed/12370818?itool=EntrezSystem2.PEntrez.Pubmed.Pubmed_ResultsPanel.Pubmed_RVDocSum&ordinalpos=1) Oncogene 21: 7266-7276.

S9. Denison SR, Callahan G, Becker NA, Phillips LA, Smith DI (2003) [Characterization of FRA6E and its potential role in autosomal recessive juvenile parkinsonism and ovarian cancer.](http://www.ncbi.nlm.nih.gov/pubmed/12874785?itool=EntrezSystem2.PEntrez.Pubmed.Pubmed_ResultsPanel.Pubmed_RVDocSum&ordinalpos=1) Genes Chromosomes Cancer 38: 40-52.

S10. Zlotorynski E, Rahat A, Skaug J, Ben-Porat N, Ozeri E et al. (2003) [Molecular basis for expression of common and rare fragile sites.](http://www.ncbi.nlm.nih.gov/pubmed/14517285?itool=EntrezSystem2.PEntrez.Pubmed.Pubmed_ResultsPanel.Pubmed_RVDocSum&ordinalpos=2) Mol Cell Biol 23: 7143-7151.

S11. Helmrich A, Stout-Weider K, Matthaei A, Hermann K, Heiden T et al. (2007) [Identification of the human/mouse syntenic common fragile site FRA7K/Fra12C1--relation of FRA7K and other human common fragile sites on chromosome 7 to evolutionary breakpoints.](http://www.ncbi.nlm.nih.gov/pubmed/17039484?itool=EntrezSystem2.PEntrez.Pubmed.Pubmed_ResultsPanel.Pubmed_RVDocSum&ordinalpos=1) Int J Cancer 120: 48-54.

S12. Hellman A, Zlotorynski E, Scherer SW, Cheung J, Vincent JB et al. (2002) [A role for common fragile site induction in amplification of human oncogenes.](http://www.ncbi.nlm.nih.gov/pubmed/12086891?itool=EntrezSystem2.PEntrez.Pubmed.Pubmed_ResultsPanel.Pubmed_RVDocSum&ordinalpos=9) Cancer Cell 1: 89-97.

S13. Ciullo M, Debily MA, Rozier L, Autiero M, Billault A et al. (2002) Hum. Mol. Genet 11: 2887-2894.

S14. Sawińska M, Schmitt JG, Sagulenko E, Westermann F, Schwab M et al. (2007) [Novel aphidicolin-inducible common fragile site FRA9G maps to 9p22.2, within the C9orf39 gene.](http://www.ncbi.nlm.nih.gov/pubmed/17668870?itool=EntrezSystem2.PEntrez.Pubmed.Pubmed_ResultsPanel.Pubmed_RVDocSum&ordinalpos=2) Genes Chromosomes Cancer 46: 991-999.

S15. Callahan G, Denison SR, Phillips LA, Shridhar V, Smith DI (2003) [Characterization of the common fragile site FRA9E and its potential role in ovarian cancer.](http://www.ncbi.nlm.nih.gov/pubmed/12555072?itool=EntrezSystem2.PEntrez.Pubmed.Pubmed_ResultsPanel.Pubmed_RVDocSum&ordinalpos=2) Oncogene 22: 590-601.

S16. Bester AC, Schwartz M, Schmidt M, Garrigue A, Hacein-Bey-Abina S et al. [Fragile sites are preferential targets for integrations of MLV vectors in gene therapy.](http://www.ncbi.nlm.nih.gov/pubmed/16511518?itool=EntrezSystem2.PEntrez.Pubmed.Pubmed_ResultsPanel.Pubmed_RVDocSum&ordinalpos=4) (2007) Gene Ther 13: 1057-109

S17. Reshmi SC, Huang X, Schoppy DW, Black RC, Saunders WS et al. (2007) [Relationship between FRA11F and 11q13 gene amplification in oral cancer.](http://www.ncbi.nlm.nih.gov/pubmed/17099871?itool=EntrezSystem2.PEntrez.Pubmed.Pubmed_ResultsPanel.Pubmed_RVDocSum&ordinalpos=1) Genes Chromosomes Cancer 46: 143154.

S18. Savelyeva L, Sagulenko E, Schmitt JG, Schwab M (2006) [The neurobeachin gene spans the common fragile site FRA13A.](http://www.ncbi.nlm.nih.gov/pubmed/16244873?itool=EntrezSystem2.PEntrez.Pubmed.Pubmed_ResultsPanel.Pubmed_RVDocSum&ordinalpos=5) Hum Genet 118: 551-558.

S19. Mangelsdorf M, Ried K, Woollatt E, Dayan S, Eyre H et al. (2000) [Chromosomal fragile site FRA16D and DNA instability in cancer.](http://www.ncbi.nlm.nih.gov/pubmed/10749140?itool=EntrezSystem2.PEntrez.Pubmed.Pubmed_ResultsPanel.Pubmed_RVDocSum&ordinalpos=2) Cancer Res 60: 1683-1689.

S20. Debacker K, Kooy RF (2007) [Fragile sites and human disease.](http://www.ncbi.nlm.nih.gov/pubmed/17567780?itool=EntrezSystem2.PEntrez.Pubmed.Pubmed_ResultsPanel.Pubmed_RVDocSum&ordinalpos=2) Hum Mol Genet 16: R150.

S21. Arlt MF, Miller DE, Beer DG, Glover TW (2002) Genes Chromosomes Cancer 33: 82-92.
